# The Cutting Edge of Gerontological Scholarship: The Gerontologist’s Editor’s Choice Symposium

**DOI:** 10.1093/geroni/igaf122.292

**Published:** 2025-12-31

**Authors:** Joseph Gaugler, Joseph Gaugler

## Abstract

The mission of The Gerontologist is to publish and disseminate “applied, multidisciplinary research and analysis on social issues related to human aging. It informs the broad community of disciplines and professions involved in understanding the aging process and providing care to older people.” In addition, the journal encourages “cutting-edge, conceptually-oriented work that addresses inequities in health, mental health, social status, and justice in late life as well as intersections among these areas with applicability to real-world-settings.” This symposium will highlight some of the recent and best examples of gerontological scholarship in The Gerontologist by highlighting select Editor’s Choice articles published in the past year. John Puxty, MbCbB, FRCP(C), Sarah Webster, MHS, and colleagues will share findings from a study that developed and validated a survey tool to evaluate Age-Friendly Community Initiatives, which will help researchers, members, and policymakers better discern the extent of age-friendliness in their communities. Jennifer Vincenzo, PhD, MPH, PT, and colleagues will present an implementation study of the Stopping Elderly Accidents, Deaths, and Injuries (STEADI) Initiative across 34 outpatient rehabilitation clinics serving older adults in a large healthcare system. Dr. Raina Croff and colleagues will describe the feasibility and health effects of a pilot triadic walking program for older Black adults with mild cognitive impairment that emphasizes culturally-centered, celebratory social reminiscence. Nikki-Anne Wilson, PhD, and Kaarin Anstey, PhD, argue for increased attention on barriers to behavior changes to reduce dementia risk in order to avoid “lifestyle” stigma.

